# The Native *Wolbachia* Endosymbionts of *Drosophila melanogaster* and *Culex quinquefasciatus* Increase Host Resistance to West Nile Virus Infection

**DOI:** 10.1371/journal.pone.0011977

**Published:** 2010-08-05

**Authors:** Robert L. Glaser, Mark A. Meola

**Affiliations:** 1 Wadsworth Center, New York State Department of Health, Albany, New York, United States of America; 2 Department of Biomedical Sciences, University at Albany, State University of New York, Albany, New York, United States of America; Nanyang Technological University, Singapore

## Abstract

**Background:**

The bacterial endosymbiont *Wolbachia pipientis* has been shown to increase host resistance to viral infection in native *Drosophila* hosts and in the normally *Wolbachia*-free heterologous host *Aedes aegypti* when infected by *Wolbachia* from *Drosophila melanogaster* or *Aedes albopictus*. *Wolbachia* infection has not yet been demonstrated to increase viral resistance in a native *Wolbachia*-mosquito host system.

**Methodology/Principal Findings:**

In this study, we investigated *Wolbachia*-induced resistance to West Nile virus (WNV; *Flaviviridae*) by measuring infection susceptibility in *Wolbachia*-infected and *Wolbachia*-free *D. melanogaster* and *Culex quinquefasciatus*, a natural mosquito vector of WNV. *Wolbachia* infection of *D*. *melanogaster* induces strong resistance to WNV infection. *Wolbachia*-infected flies had a 500-fold higher ID_50_ for WNV and produced 100,000-fold lower virus titers compared to flies lacking *Wolbachia*. The resistance phenotype was transmitted as a maternal, cytoplasmic factor and was fully reverted in flies cured of *Wolbachia*. *Wolbachia* infection had much less effect on the susceptibility of *D*. *melanogaster* to Chikungunya (*Togaviridae*) and La Crosse (*Bunyaviridae*) viruses. *Wolbachia* also induces resistance to WNV infection in *Cx. quinquefasciatus*. While *Wolbachia* had no effect on the overall rate of peroral infection by WNV, *Wolbachia*-infected mosquitoes produced lower virus titers and had 2 to 3-fold lower rates of virus transmission compared to mosquitoes lacking *Wolbachia*.

**Conclusions/Significance:**

This is the first demonstration that *Wolbachia* can increase resistance to arbovirus infection resulting in decreased virus transmission in a native *Wolbachia*-mosquito system. The results suggest that *Wolbachia* reduces vector competence in *Cx. quinquefasciatus*, and potentially in other *Wolbachia*-infected mosquito vectors.

## Introduction

*Wolbachia pipientis* is an intracellular, α-proteobacterial symbiont that infects a wide variety of invertebrates, including insects, spiders, mites, isopod crustaceans, and filarial nematodes [1], [2], [3], [4], [5]. It was first identified in the mosquito *Culex pipiens* and has been most studied for the broad range of reproductive phenotypes that it induces in its various hosts, including cytoplasmic incompatibility, feminization, parthenogenesis, and male killing [6], [7]. These reproductive phenotypes help to ensure the bacterium's persistence in the host population and have made *Wolbachia* a highly successful symbiont, infecting up to 66% of arthropod species [8], [9].

*Wolbachia* has been shown to infect at least 19 species of fruit flies of the genus *Drosophila* [10], [11], [12], [13], [14]. In some species, like *D*. *simulans*, *Wolbachia* causes robust and complex patterns of cytoplasmic incompatibility, while in other species, like *D*. *melanogaster*, reproductive phenotypes are generally weak or absent [14], [15], [16], [17], [18], [19], [20], [21], [22]. Despite the lack of a strong reproductive phenotype, *Wolbachia* infection of *D*. *melanogaster* is nonetheless widespread [18], [22], [23], [24]. This paradox suggests that non-reproductive phenotypes probably confer a fitness advantage to *Wolbachia*-infected *D*. *melanogaster*, thereby explaining the observed persistence of *Wolbachia* infection. A variety of non-reproductive phenotypes have additionally been identified in *Wolbachia*-infected *D*. *melanogaster*, including effects on behavior, viability, insulin signaling, and iron homeostasis [25], [26], [27], [28], [29], [30], [31], [32], [33]. While the magnitude of most of these *Wolbachia*-dependent phenotypes is generally modest and frequently variable, some of these could, nonetheless, provide *Wolbachia*-infected flies with a selective fitness advantage [32], [33], [34].

*Wolbachia* infection of *D*. *melanogaster* has also been shown to increase the fly's resistance to some viral infections, resulting in infections with lower virus titers and less associated pathology [35], [36]. The resistance phenotype appears to be limited to RNA viruses, with the strength of resistance varying substantially among the viruses tested thus far. For example, infection of *D*. *melanogaster* by Drosophila C virus or cricket paralysis virus (DCV and CrPV; *Dicistroviridae*) is strongly suppressed by *Wolbachia* infection with titers of DCV being reduced up to 10,000-fold [35], [36]. In contrast, infection by Flock House virus (FHV; *Nodaviridae*) is not inhibited by *Wolbachia* at the level of virus replication, yet the pathology associated with FHV infection is strongly reduced [35]. For natural viral infections of *D*. *melanogaster* that cause pathology, such as DCV, resistance to viral infection would clearly confer *Wolbachia*-infected *D*. *melanogaster* a significant fitness advantage in the face of viral infection.

*Wolbachia* from *D. melanogaster* can also confer resistance to viral infection in a heterologous host. *Wolbachia* strain *w*MelPop, which normally infects *D. melanogaster*, lacks normal replication control, resulting in significant pathology and a shortened lifespan in the fly [37]. Infection of the normally *Wolbachia*-free mosquito *Aedes aegypti* with a mosquito-adapted strain of *w*MelPop produces mosquitoes with both a shortened lifespan and increased resistance to viral infection [38], [39]. In addition, infection of *Ae. aegypti* with *Wolbachia* from *Aedes albopictus* also increases viral resistance [40]. These results clearly demonstrate that *Wolbachia* can increase viral resistance when infecting a heterologous mosquito host. It is less clear, however, whether *Wolbachia* ever increases viral resistance when infecting their native mosquito hosts. To date, this question has only been addressed in *Ae*. *albopictus*, and no increase in susceptibility to dengue virus (DENV; *Flaviviridae*) or Chikungunya virus (CHIKV; *Togaviridae*) infection was observed in *Ae*. *albopictus* mosquitoes cured of their normal *Wolbachia* symbionts, suggesting that *Wolbachia* infection does not increase viral resistance in this native *Wolbachia*-mosquito system [40], [41].

To investigate this question further, we looked for *Wolbachia*-induced increases in resistance to infection by West Nile virus (WNV; *Flaviviridae*) in *D. melanogaster* and in the southern house mosquito *Culex quinquefasciatus*, a natural vector of WNV. In both cases, we looked for viral resistance induced by the *Wolbachia* strain that naturally infects each species. We demonstrate that *Wolbachia* infection of *D*. *melanogaster* increases resistance to infection by WNV and that this protective effect is relatively specific to WNV compared to other arboviruses. We further demonstrate that *Wolbachia* also increases resistance to WNV infection in the mosquito *Cx. quinquefasciatus*. While more modest than the level of resistance observed in flies, the resistance phenotype in *Cx. quinquefasciatus* was sufficient to significantly reduce the proportion of infected mosquitoes that transmitted virus during feeding.

## Results

### *Wolbachia* increases resistance to WNV infection in *D. melanogaster*

Our previous studies have shown that mutations in the RNAi pathway of *D. melanogaster* increase susceptibility of flies to infection by WNV [42]. During the course of these earlier studies, we unexpectedly discovered that RNAi mutant strain *Ago2^414^* had the opposite phenotype, being highly resistant to WNV infection (Fig. S1; Text S1). Further genetic analysis determined that the resistance phenotype was caused by a dominant, maternally transmitted, cytoplasmic factor and not the nuclear genotype of *Ago2^414^* flies (Figs. S2 and S3; Text S1). Maternal cytoplasmic transmission of the phenotype combined with the known ability of the bacterial endosymbiont *Wolbachia pipientis* to induce resistance to viral infection in flies implicated *Wolbachia* as the cause of the WNV resistance phenotype [35], [36]. We directly tested this hypothesis and found that the *Ago2^414^* flies were, in fact, infected with *Wolbachia* (Fig. S4; Text S1) and that curing the flies of *Wolbachia* infection reverted the resistance phenotype, producing flies fully susceptible to WNV infection (Fig. S5; Text S1). These results strongly support the conclusion that *Wolbachia* infection causes the WNV resistance phenotype seen in the *Ago2^414^* flies.

**Figure 1:**
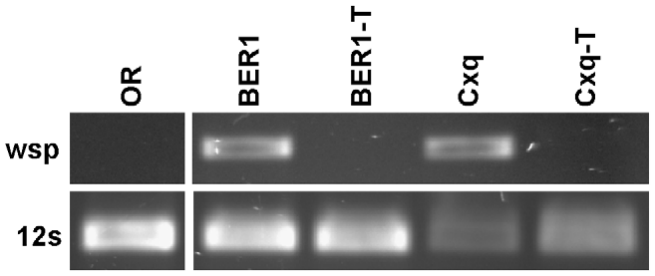
*Wolbachia* status of *D*. *melanogaster* and *Cx. quinquefasciatus* strains analyzed for susceptibility to arbovirus infection. DNA was isolated from *D*. *melanogaster* strains Oregon R (OR) and BER1, and from *Cx. quinquefasciatus* (Cxq). Tetracycline-treated strains lacking *Wolbachia* sequences are indicated by the suffix -T. DNA sequences corresponding to the *wsp* gene of *Wolbachia* and the *12S* mitochondrial gene were identified by PCR.

**Figure 2:**
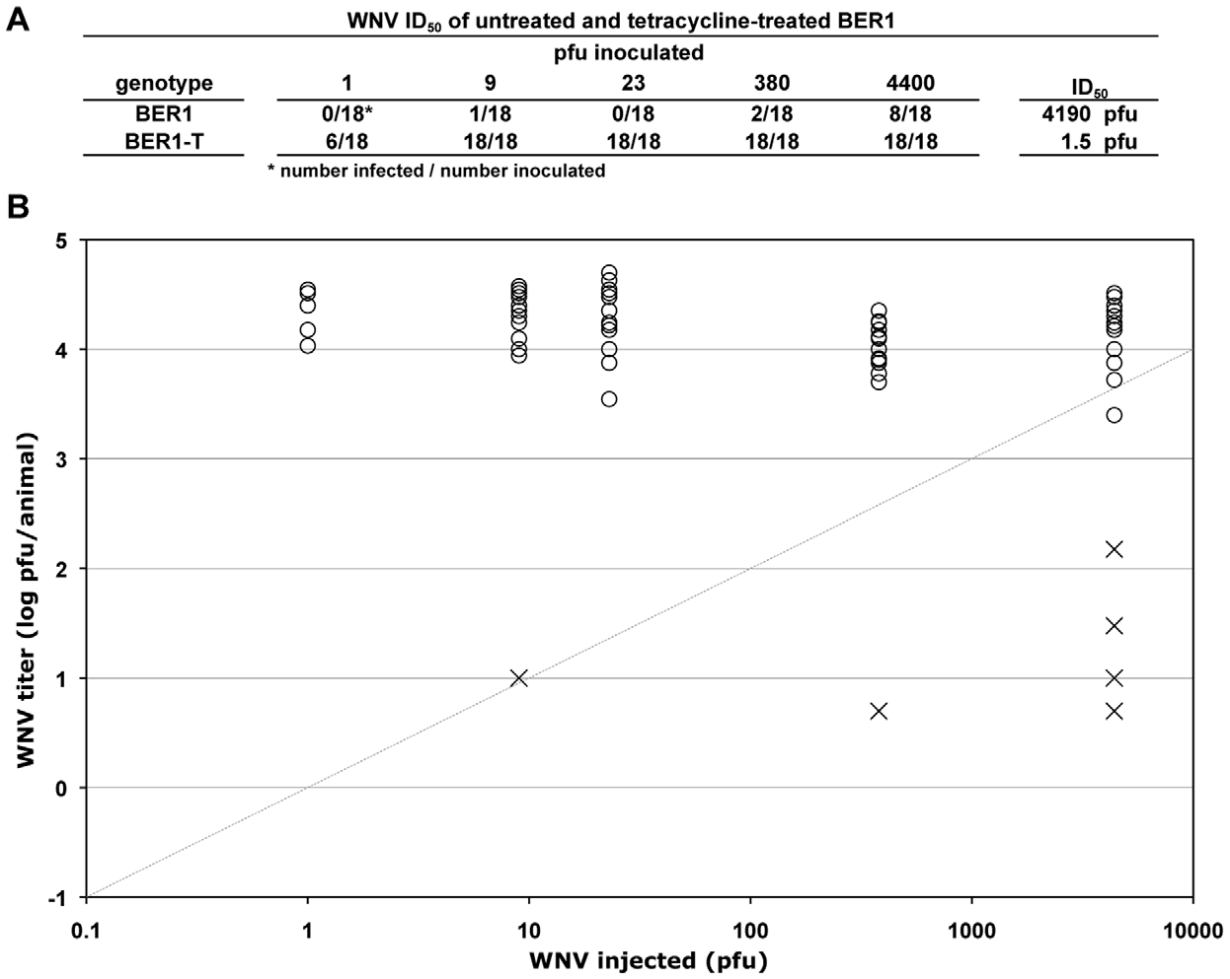
*Wolbachia*-induced resistance to WNV infection in wild-type *D*. *melanogaster* strain BER1. The indicated pfu of WNV was injected into *Wolbachia*(+) BER1 and *Wolbachia*(−) BER1-T strains of *D*. *melanogaster*. Seven days after inoculation, the titer of WNV in each fly was measured by plaque assay. (A) The fraction of flies that became infected for each genotype at each concentration of virus, and the ID_50_ value for each genotype as calculated from those data, are shown. (B) The titers of WNV in infected *Wolbachia*(+) BER1 (X) and *Wolbachia*(−) BER1-T flies (O) are shown. The grey diagonal line indicates the amount of WNV inoculated per fly. The limit of detection of the plaque assay was 5 pfu/animal.

To assess whether resistance to WNV infection is a general phenotype of *Wolbachia*-infected *D*. *melanogaster* and not a phenotype unique to the *Ago2^414^* mutant strain, we measured susceptibility to WNV infection in BER1 flies, a wild type strain of flies naturally infected with *Wolbachia*, and in tetracycline-treated BER1-T flies cured of *Wolbachia* infection (Fig. 1). *Wolbachia*(+) BER1 flies were found to be resistant to infection, with an ID_50_ for WNV of 4190 plaque forming units (pfu) and low titers of virus in infected flies (Fig. 2). In contrast, *Wolbachia*(-) BER1-T flies were susceptible to infection, with an ID_50_ for WNV of 1.5 pfu and consistently high titers of virus in all infected flies (Fig. 2). The results for the BER1 flies paralleled what was observed for the *Ago2^414^* flies (*cf.* Figs. 2 and S5), suggesting that resistance to WNV infection is a general feature of *Wolbachia*-infected *D*. *melanogaster*.

**Figure 3:**
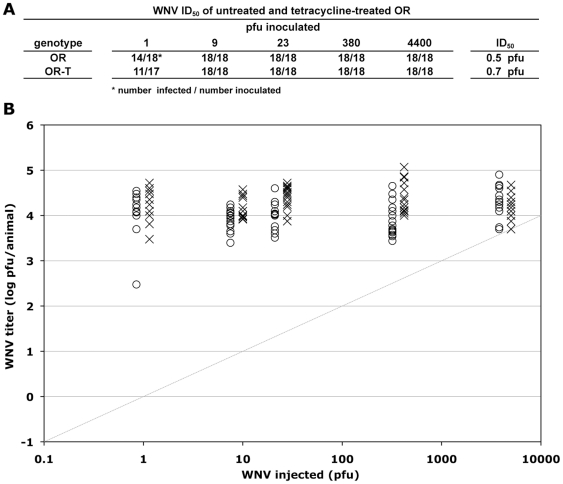
Tetracycline treatment of *Wolbachia*(−) Oregon R flies had little effect on susceptibility to WNV infection. The indicated pfu of WNV was injected into untreated (OR) and tetracycline-treated (OR-T) Oregon R flies. Seven days after inoculation, the titer of WNV in each fly was measured by plaque assay. (A) The fraction of flies that became infected for each genotype at each concentration of virus, and the ID_50_ value for each genotype as calculated from those data, are shown. (B) The titers of WNV in untreated (X) and tetracycline-treated flies (O) are shown. The grey diagonal line indicates the amount of WNV inoculated per fly. The limit of detection of the plaque assay was 25 pfu/animal.

To address the possibility that tetracycline treatment itself caused the observed increase in WNV susceptibility independent of the loss of *Wolbachia* infection, we treated wild-type Oregon R flies (OR), which are not infected with *Wolbachia* (Fig. 1), with tetracycline, and then compared WNV susceptibility in untreated OR and tetracycline-treated OR-T flies (Fig. 3). In contrast to the dramatically higher susceptibility observed for tetracycline-treated BER1-T flies, tetracycline treatment of OR flies actually reduced susceptibility (Fig. 3). Their ID_50_ values were 0.5 versus 0.7 for OR and OR-T flies, respectively, and virus titers in the OR-T flies were 1.7-fold lower when averaged across all the virus titers tested (Fig. 3; *p*<0.001, t-test). Thus, tetracycline treatment itself does not cause increased viral susceptibility, supporting the conclusion that it is the loss of *Wolbachia* that is responsible for the observed increase in WNV susceptibility.

### WNV is more sensitive to *Wolbachia-*induced resistance than CHIKV or LACV

**Figure 4:**
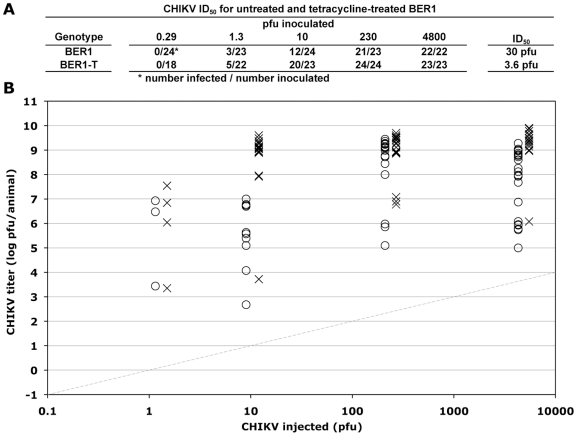
*Wolbachia*-induced resistance to CHIKV infection in *D*. *melanogaster*. The indicated pfu of CHIKV was injected into *Wolbachia*(+) BER1 and *Wolbachia*(−) BER1-T flies. Seven days after inoculation, the titer of CHIKV in each fly was measured by plaque assay. (A) The fraction of flies that became infected for each genotype at each concentration of virus, and the ID_50_ value for each genotype as calculated from those data, are shown. (B) The titers of CHIKV in infected *Wolbachia*(+) BER1 (O) and *Wolbachia*(−) BER1-T flies (X) are shown. The grey diagonal line indicates the amount of CHIKV inoculated per fly. The limit of detection of the plaque assay was 25 pfu/animal.

Mosquito-vectored arboviruses that cause human zoonotic disease include viruses from the *Flaviviridae* (including WNV), *Togaviridae*, and *Bunyaviridae* families. To assess the relative strength of *Wolbachia*-induced viral resistance against representative *Togaviridae* and *Bunyaviridae* viruses, we compared the susceptibility of BER1 and BER1-T flies to infection with Chikungunya virus (CHIKV; *Togaviridae*) and La Crosse virus (LACV; *Bunyaviridae*). CHIKV proliferates robustly after inoculation into *D*. *melanogaster* (Fig. 4). The ID_50_ for CHIKV in *Wolbachia*(+) BER1 flies was 30 pfu, and the average titer of virus in infected animals for the various concentrations of CHIKV inoculated ranged from 6.4 to 9.1 log_10_ pfu/animal (Fig. 4). *Wolbachia*(−) BER1-T flies were more susceptible to CHIKV infection than were the BER1 flies. The ID_50_ for the BERT1-T flies was 3.6 pfu, 8-fold lower than that for BER1 flies, and the average titer of virus in infected BER1-T flies was higher at every concentration of virus tested, averaging 4.2-fold higher when calculated across all inoculation doses combined (*p*<1×10^−7^, t-test). Thus, CHIKV infection of BER1 flies is inhibited by *Wolbachia*, but the effect is weaker than observed for WNV; specifically, the ID_50_ decreased by 8-fold (CHIKV) versus 2793-fold (WNV) and the virus titer increased by 4.2-fold (CHIKV) versus 918-fold (WNV) when averaged across all infected animals (*cf*. Figs. 2 and 4).

**Figure 5:**
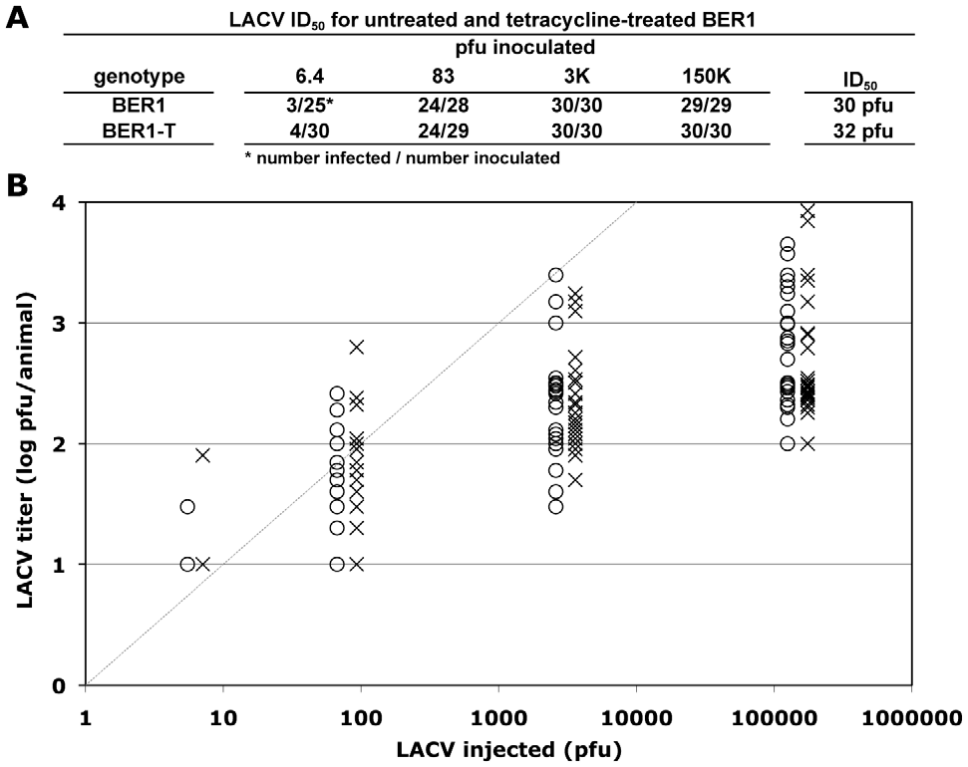
*Wolbachia* did not increase resistance to LACV infection in *D*. *melanogaster*. The indicated pfu of LACV was injected into *Wolbachia*(+) BER1 and *Wolbachia*(−) BER1-T flies, and seven days after inoculation, the titer of LACV was measured in each fly by plaque assay. (A) The fraction of flies that became infected for each genotype at each concentration of virus, and the ID_50_ value for each genotype as calculated from those data, are shown. (B) The titers of LACV in infected *Wolbachia*(+) BER1 (O) and *Wolbachia*(−) BER1-T flies (X) are shown. The grey diagonal line indicates the amount of CHIKV inoculated per fly. The limit of detection of the plaque assay was 10 pfu/animal.

LACV replicates much less robustly in *D*. *melanogaster* than does either WNV or CHIKV (Fig. 5). The low infectivity of LACV in flies was anticipated. Tahyna virus has been shown previously to be able to infect tissue culture cells of *D*. *melanogaster,* producing a persistent infection, but releasing significantly less virus than cells infected with WNV or CHIKV [43]. Because Tahyna virus and LACV are both strains of California encephalitis virus, we could anticipate that the two viruses would behave comparably in *D*. *melanogaster*. The ID_50_ for LACV in *Wolbachia*(+) BER1 flies was 30 pfu, and the average titer of virus in infected animals ranged from 1.5 to 3 log_10_ pfu/animal for the various concentrations of LACV that were inoculated (Fig. 5). At the lower titers of inoculum, more virus was produced during infection than was injected, supporting the conclusion that LACV does cause low levels of infection in flies, comparable to what has been reported for tissue culture cells [43]. In contrast to the results found for WNV and CHIKV, susceptibility to LACV was essentially unchanged between *Wolbachia*(+) BER1 and *Wolbachia*(−) BER1-T flies. The ID_50_ for the latter was 32 pfu, and the average titer of virus in infected animals for the various concentrations of LACV tested was not significantly different from the titer determined in *Wolbachia*(+) BER1 flies (Fig. 5). The limited extent of the infection caused by LACV does not allow us to draw strong conclusions from these data, the results, nonetheless, suggest that *Wolbachia* infection of *D*. *melanogaster* does not inhibit infection by LACV.

### *Wolbachia* inhibits WNV infection and reduces vector competence in *Cx. quinquefasciatus*

*Wolbachia* that normally infect mosquito vectors of WNV are not likely to inhibit viral infection to the same degree as seen in *D*. *melanogaster*. For example, North American populations of the southern house mosquito *Culex quinquefasciatus* are widely, if not universally, infected by *Wolbachia*, yet can be infected by and transmit WNV in the lab, and are found infected by WNV in the field, suggesting that *Cx. quinquefasciatus* is a natural vector of WNV despite being infected by *Wolbachia* [44], [45], [46], [47], [48], [49], [50], [51], [52]. Nonetheless, we considered the possibility that *Wolbachia* might still inhibit WNV infection in *Cx. quinquefasciatus*, but less than observed in *D. melanogaster*.

A laboratory strain of *Cx. quinquefasciatus* infected with *Wolbachia* was cured of *Wolbachia* infection by treatment with tetracycline. No *Wolbachia wsp* gene sequences were detected in the mosquitoes after treatment (Fig. 4). In addition, the loss of *Wolbachia* was evidenced by the appearance of strong cytoplasmic incompatibility. Crosses between females from the treated *Wolbachia*(−) strain and males from the original *Wolbachia*(+) strain were fully infertile, while the reciprocal cross and crosses between *Wolbachia*(−) individuals were fertile (data not shown). Finally, there was no significant difference in the wet weight or wing length of newly emerged females from the *Wolbachia*(+) and *Wolbachia*(−) strains, suggesting that *Wolbachia* infection does not have a significant effect on the growth of *Cx. quinquefasciatus* (data not shown).

To determine if *Wolbachia* infection of *Cx. quinquefasciatus* affects susceptibility of the mosquitoes to WNV, we fed *Wolbachia*(+) and *Wolbachia*(−) mosquitoes a blood meal containing WNV, and determined the frequencies of infected mosquitoes, of virus dissemination into the legs, and of virus transmission in the saliva at 5, 7, and 14 days post blood meal (Table 1). Since all *Cx*. *quinquefasciatus* mosquitoes are normally infected with *Wolbachia*, we were unable to test a naturally *Wolbachia*-free population of *Cx*. *quinquefasciatus* for nonspecific affects of tetracycline treatment, as was done for *D. melanogaster* (Fig. 3). Instead, we repeated the experiment at five and fourteen generations after tetracycline treatment, reasoning that any nonspecific phenotypes caused by antibiotic toxicity would likely be transient and fail to persist for more than a few generations. The *Cx. quinquefasciatus* colony recovered rapidly after tetracycline treatment and was easily expanded to normal numbers of animals within two generations without any obvious reductions in fertility, fecundity, or viability.

**Table 1:** Vector competence of untreated and tetracycline-treated *Cx*. *quinquefasciatus*.

		5th generation after treatment			14th generation after treatment		
dpm	*Wolbachia*	I	D	T	I	D	T
5	+	23/25 (92)*	2/25 (8)	1/25 (4)	15/17 (88)	2/17 (12)	1/17 (6)
	−	28/29 (97)	9/29 (31)	4/29 (14)	19/21 (90)	6/21 (29)	2/21 (10)
7	+	23/23 (100)	2/23 (9)	0/23 (0)	15/17 (88)	4/17 (24)	1/17 (6)
	−	28/28 (100)	8/28 (29)	6/28 (21)	23/23 (100)	8/23 (35)	4/23 (17)
14	+	25/25 (100)	6/25 (24)	2/25 (8)	14/17 (82)	4/17 (24)	3/17 (18)
	−	23/23 (100)	8/23 (35)	4/23 (17)	20/20 (100)	12/20 (60)	8/20 (40)

dpm, days post blood meal; I, infected body; D, disseminated to legs; T, transmitted to saliva.

*number of mosquitoes positive for virus/number assayed (percent).

At the relatively high titer of virus added to the blood meal, most of both the *Wolbachia*(+) and *Wolbachia*(−) mosquitoes became infected (Table 1). The frequency of infection of *Wolbachia*(+) and *Wolbachia*(−) mosquitoes were similar even when the overall frequency of infection was reduced by adding less virus to the blood meal or by culturing blood-fed mosquitoes at lower temperatures (data not shown). In contrast to the similarity in overall infection frequency, the frequencies of virus dissemination into the legs and of virus transmission in the saliva were higher in the *Wolbachia*(−) mosquitoes at all time points tested at both five and fourteen generations (Table 1). The increase was between 2 and 3-fold at most time points. The fact that dissemination and transmission rates for the *Wolbachia*(−) mosquitoes were higher at all time points is highly significant (*p*<0.001; binomial probability), and the fact that the same increases were observed both five and fourteen generations after antibiotic treatment suggests that the increased rates of WNV dissemination and transmission are a permanent phenotypic consequence of removing *Wolbachia* from *Cx*. *quinquefasciatus* mosquitoes rather than being a transient artifact caused by antibiotic treatment.

**Figure 6:**
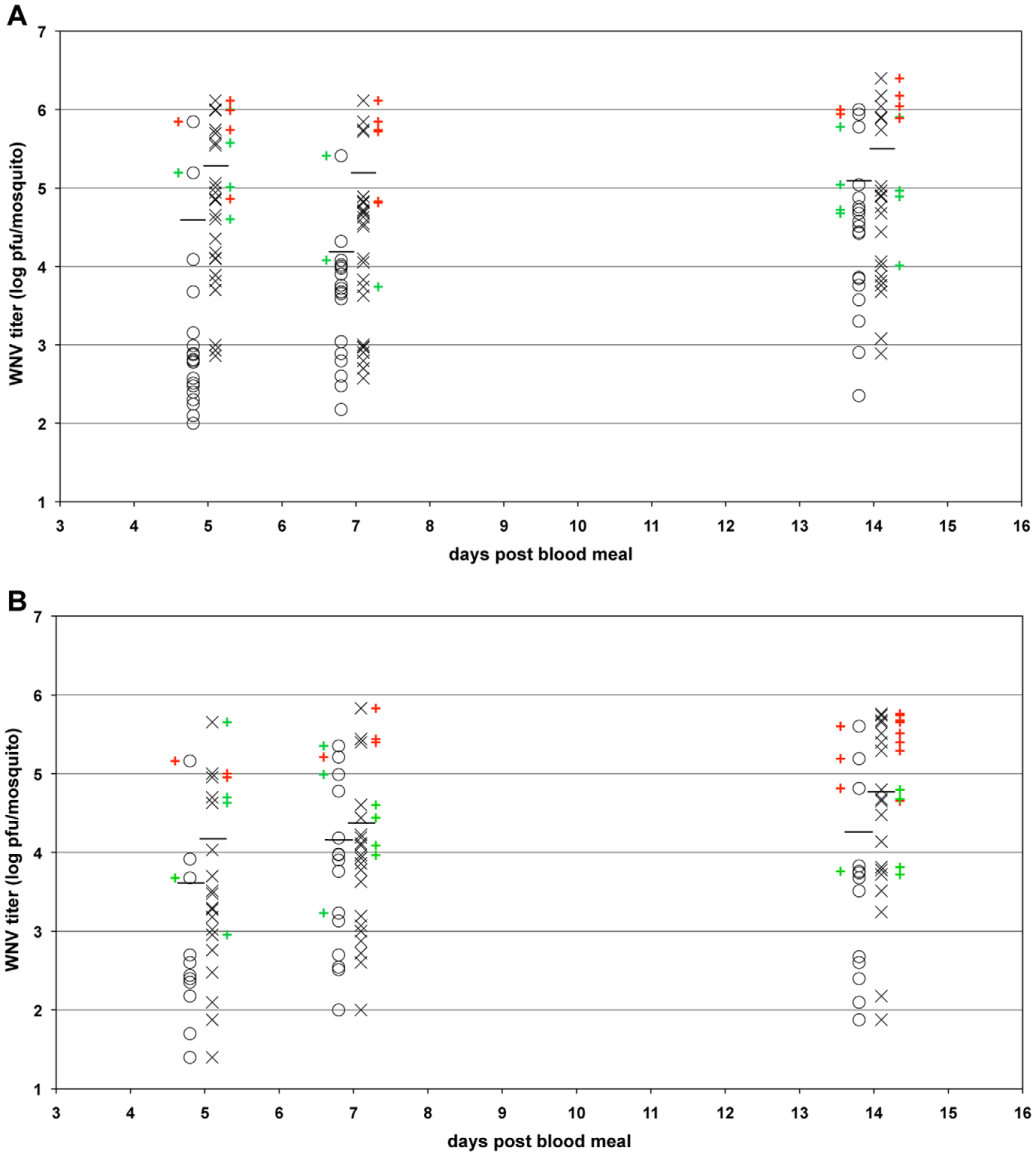
Titers of WNV in infected *Wolbachia*(+) and *Wolbachia*(−) *Cx. quinquefasciatus* mosquitoes. *Wolbachia*(+) and *Wolbachia*(−) *Cx. quinquefasciatus* mosquitoes were fed a blood meal containing WNV either five generations (A) or fourteen generations (B) after tetracycline treatment. Titers of WNV in infected *Wolbachia*(+) mosquitoes (O) and in infected *Wolbachia*(−) mosquitoes (X) were measured by plaque assay 5, 7, and 14 days post blood meal. The average virus titer is indicated by a horizontal line. Mosquitoes in which virus had disseminated only to the legs (green) or that had disseminated to the legs and was transmitted in the saliva (red) are indicated by colored plus signs located next to the virus titer for that same mosquito.

Since the overall rate of infection did not differ between *Wolbachia*(+) and *Wolbachia*(−) mosquitoes, it was unclear if the increased rate of virus dissemination and transmission observed in *Wolbachia*(−) mosquitoes was a consequence of increased susceptibility of the mosquitoes to WNV infection (Table 1). To address this question, we measured the titers of WNV in the bodies of all the infected mosquitoes presented in Table 1 (Figure 6). While virus titers varied widely in both the *Wolbachia*(+) and *Wolbachia*(−) mosquitoes, the average titer was higher in *Wolbachia*(−) mosquitoes at all time points tested (Figure 6). The higher average titers in *Wolbachia*(−) mosquitoes, however, were only significant at five generations, but not at fourteen generations after treatment (*p*<0.00001 and *p*>0.05, respectively; ANOVA). More importantly, the probability of virus dissemination and transmission was strongly correlated with virus titer in both the five generation and fourteen generation experiments (*p*<0.0001; χ^2^ test). Infections producing titers beyond a threshold of about 4.5 log_10_ pfu/mosquito were likely to result in virus dissemination, and in infections with the highest titers, virus transmission. This correlation is particularly apparent when dissemination and transmission status is compared with virus titer for each mosquito (green and red pluses in Fig. 6). Most importantly, there was clearly a greater proportion of *Wolbachia*(−) mosquitoes compared to *Wolbachia*(+) mosquitoes having the highest virus titers at each time point tested. The increase in the number of *Wolbachia*(−) mosquitoes with the highest virus titers correlates with, and can explain, the 2 to 3-fold higher rates of virus dissemination and transmission observed for the *Wolbachia*(−) mosquitoes (*cf*. Fig. 6 and Table 1). These results support the conclusion that *Wolbachia* normally inhibits WNV infection in *Cx. quinquefasciatus* mosquitoes, limiting virus titers in infected animals and reducing the probability that an infected mosquito will transmit virus during feeding. Curing *Cx. quinquefasciatus* of *Wolbachia* infection, as reported here, removed that inhibition, resulting in a greater proportion of infected mosquitoes developing the high virus titers necessary for virus dissemination and transmission.

## Discussion

This is the first report of *Wolbachia*-induced resistance to arbovirus infection in a native *Wolbachia*-mosquito system. While the experiments reported here were done on a laboratory colony, the results raise the possibility that *Wolbachia* infection could impact vector competence of *Cx. quinquefasciatus* in the field. For example, vector competence is known to vary between individuals and between different populations of *Cx. quinquefasciatus* [49], [50]. Similarly, *Wolbachia* infection densities can vary 100-fold between individual field-collected *Cx*. *pipiens*, a closely related mosquito species [53], [54]. Furthermore, *Wolbachia* infection levels are dynamic and can be sensitive to both environmental factors, such as temperature, and host genetic factors that vary between populations [54], [55], [56], [57], [58], [59]. So, if the strength of *Wolbachia*-induced viral resistance is sensitive to the differences in *Wolbachia* levels that occur between mosquitoes, as some evidence suggests, then vector competence in *Cx. quinquefasciatus* could potentially be modulated indirectly through environmental and genetic factors that modify levels of *Wolbachia* [39], [60]. In addition to differences in vector competence within a species, *Wolbachia* infection might also contribute to differences in vector competence between species. For example, *Cx*. *quinquefasciatus*, which is infected with *Wolbachia*, is generally less susceptible to WNV infection than *Culex tarsalis*, which is not infected with *Wolbachia* [50]. Our results suggest that the difference in *Wolbachia* infection between these two species could contribute, at least in part, to the observed difference in vector competence.

It is unclear to what extent *Wolbachia* might inhibit arbovirus susceptibility and vector competence in other species of mosquitoes naturally infected by *Wolbachia*. Given that the strength of *Wolbachia*-induced viral resistance in *Drosophila* is known to be dependent on both virus type and *Wolbachia* strain, our observation of *Wolbachia*-induced resistance to WNV in *Cx. quinquefasciatus* is not necessarily applicable to other specific *Wolbachia*-mosquito-arbovirus interactions [35], [36], [60]. In the one other mosquito species in which *Wolbachia*-induced viral resistance has been directly tested, *Ae*. *albopictus*, no increase in susceptibility to either DENV or CHIKV infection was observed in mosquitoes cured of *Wolbachia*, suggesting that the *Wolbachia* that normally infect *Ae*. *albopictus* do not increase resistance to viral infection [40], [41]. Even this conclusion, however, may not be generally applicable to all *Ae*. *albopictus* mosquitoes, since *Wolbachia* levels in somatic tissues can vary significantly between different strains of *Ae*. *albopictus*, from relatively abundant to undetectable, potentially impacting whether a resistance phenotype is observed [59].

*Wolbachia* infection in *Cx. quinquefasciatus* inhibits the dissemination and transmission of WNV but not the overall frequency of infection (Table 1). In the closely related species *Culex pipiens*, *Wolbachia* levels are much lower in the midgut than in other somatic tissues [59]. If *Wolbachia* is distributed the same way in *Cx. quinquefasciatus*, then *Wolbachia* may not be present to inhibit infection in the midgut epithelial cells where viral infection begins (assuming that the mechanism of *Wolbachia*-induced resistance is cell autonomous), resulting in the overall rate of viral infection being independent of the presence or absence of *Wolbachia*. As the WNV infection spread, however, the virus would encounter tissues containing *Wolbachia*, and therefore more resistant to infection, resulting in the lower virus titers and decreased rates of virus dissemination and transmission that were observed. The degree of overlap in the tissue distribution of *Wolbachia* and viral pathogen is likely to be an important determinant in the extent to which *Wolbachia* can inhibit vector competence in any given *Wolbachia*-mosquito-arbovirus interaction [39]. Finally, *Wolbachia*-induced resistance to WNV may be weaker in *Cx. quinquefasciatus* than in *D*. *melanogaster* simply because levels of *Wolbachia* are lower in the somatic tissues of *Cx. quinquefasciatus* than in *D*. *melanogaster*. Preliminary comparison of *Wolbachia* levels in the two species is consistent with that conclusion (unpublished observations).

There are at least eleven major supergroups of *Wolbachia*, and until recently, all the *Wolbachia* strains shown to increase viral resistance have been supergroup A strains that normally infect *D. melanogaster* and *D. simulans* [35], [36], [39], [60], [61]. Recently, supergroup B strain *w*AlbB from *Ae*. *albopictus* was shown to increase resistance to DENV in the heterologous host *Ae*. *aegypti* [40]. This result does not necessarily mean that supergroup B strains also induce viral resistance in their native hosts, since host responses to *Wolbachia* infection are know to differ between heterologous and native hosts. For example, *w*AlbB induces the innate immune response in heterologous host *Ae*. *aegypti* but not in native host *Ae*. *albopictus* [40], [62]. The *w*Pip strain of *Wolbachia* infecting *Cx. quinquefasciatus* is a supergroup B strain of *Wolbachia*, and the results reported here are the first demonstration of viral resistance induced by a supergroup B strain of *Wolbachia* in its native host [12], [63]. The one other study that has looked for viral resistance induced by a supergroup B strain in its native host found that strain *w*No infecting *D*. *simulans* does not increase resistance against infection by either DCV or FHV [60]. They also found, however, that levels of *w*No were significantly lower than the levels of three supergroup A strains that also naturally infect *D*. *simulans* and that all increase viral resistance [60]. Our observation of a *Wolbachia*-dependent increase in viral resistance in *Cx. quinquefasciatus* demonstrates that supergroup B strains of *Wolbachia* are capable of increasing resistance to viral infection in their native hosts, and argues that the absence of *Wolbachia*-induce resistance in *w*No-infected *D*. *simulans* is more likely due to low *Wolbachia* density than to an intrinsic inability of supergroup B strains to confer a resistance phenotype on their native hosts.

Recently, both *D. melanogaster*-derived *w*MelPop and *Ae*. *albopictus*-derived *w*AlbB strains of *Wolbachia* when transferred into *Ae*. *aegypti* have been shown to significantly increase resistance of *Ae*. *aegypti* to DENV infection, with virus titers being suppressed more than 10,000-fold in both cases [39], [40]. This level of viral resistance is comparable to the strong resistance phenotype observed for WNV infection in *D*. *melanogaster* (Figs. 2, s5). The fact that both WNV and DENV are especially sensitive to *Wolbachia*-induced resistance in both native and heterologous hosts, respectively, suggests that flaviviruses, in general, may be particularly susceptible to *Wolbachia*-induced resistance and that the mechanism of resistance to flavivirus infection is the same in flies and mosquitoes. In contrast, the strength of *Wolbachia*-induced resistance to infection by the alphavirus CHIKV differed markedly between the same *D*. *melanogaster* and *A*. *aegypti Wolbachia*-host systems. In *D*. *melanogaster*, *Wolbachia*-induced resistance to CHIKV was modest and significantly lower than the resistance to WNV, while in *A*. *aegypti*, resistance to CHIKV and to DENV were equally strong (Fig. 4)[39]. The reason for the difference in the relative susceptibility of CHIKV to *Wolbachia*-induced resistance is not known, but likely arises from one of the differences between the *Wolbachia*-host systems studied, which include the hosts (*D*. *melanogaster* versus *A*. *aegypti*), the strains of *Wolbachia* (*w*Mel versus mosquito-adapted *w*MelPop), and the strains of virus (CHIKV versus CHIKV^E1-A226V^). *Wolbachia*-induced phenotypes, including viral resistance, are known to be sensitive to differences in both host species and *Wolbachia* strain [60], [64], [65].

The strength of *Wolbachia*-induced suppression of viral infection in *D*. *melanogaster* varies widely amongst those viruses that have been tested. Infections by WNV (*Flaviviridae*) and DCV (*Dicistroviridae*) are both strongly suppressed, with virus titers being reduced at least 10,000-fold for both viruses (this report)[35]. Infections by CHIKV (*Togaviridae*) and NoraV virus (picorna-like family), in contrast, are only modestly suppressed, with virus titers being reduced about 10-fold, while infections by LACV (*Bunyaviridae*), FHV (*Nodaviridae*) and IIV-6 (*Iridoviridae*) are unaffected (this report)[35]. It is not clear why WNV and DCV are both so selectively sensitive to *Wolbachia*-induced resistance. Although both are positive-sense, ssRNA viruses, other positive-sense, ssRNA viruses are less strongly affected (CHIKV, NoraV) or are not affected at all at the level of virus replication (FHV). It is also unclear whether negative-sense, ssRNA viruses like LACV and dsDNA viruses like IIV-6 will, as a general rule, be refractory to *Wolbachia*-induced resistance, given that only a single representative virus of either type has thus far been tested (this report)[35]. If these results are representative, however, then *Wolbachia*-induce resistance may be limited to a subset of positive-sense, ssRNA viruses. An elucidation of virus specificity, particularly the marked sensitivity shared by relatively dissimilar viruses like WNV and DCV, will require a better understanding of the underlying mechanism, or mechanisms, by which *Wolbachia* infection increases viral resistance.

Finally, it is noteworthy that *Wolbachia* infection of *D*. *melanogaster* inhibits WNV infection so dramatically without causing significant deleterious effects to the host. Other than resistance to viral infection, the particular *Wolbachia*-infected strains of *D. melanogaster* studied here are unremarkable, with no obvious reductions in viability, fertility, or fecundity (unpublished observations). Elucidating the underlying molecular mechanism by which *Wolbachia* inhibits viral infection in *D*. *melanogaster* could, therefore, promote the development of antiviral agents that either mimic the direct antiviral action of *Wolbachia* proteins or modulate in therapeutically useful ways the same host pathways important for viral infection. The complexity inherent in a biological system comprising the interaction of three disparate organisms - bacterial symbiont, insect host, and viral pathogen - presents significant challenges for future mechanistic studies. Such studies will be facilitated by the availability of extensive genetic and molecular tools developed for *D*. *melanogaster*.

## Materials and Methods

### Insects and tetracycline treatment

*D*. *melanogaster* were maintained on cornmeal-brewer's yeast-glucose medium at 23°C and 45% relative humidity. Wild-type *D*. *melanogaster* strains Oregon R and BER1 were obtained from the Bloomington Drosophila Stock Center. *AGO2^414^* flies were obtained from Haruhiko Siomi [66]. Flies were cured of *Wolbachia* infection by growing the flies for one generation on instant food (Carolina Biological) made with 200 µg/ml tetracycline. Removal of the *Wolbachia* was confirmed by PCR.

*Cx. quinquefasciatus* were maintained at 26°C, 50% RH with a 16∶8 L∶D photoperiod. Larvae were reared at 300 larvae/liter with a water depth of 1.5 cm and fed standardized volumes of ground koi pellets. Adults were maintained on 10% sucrose *ad libitum* and fed goose blood supplemented with 2.5% sucrose for egg laying. The *Wolbachia*(+) *Cx. quinquefasciatus* colony was established from mosquitoes obtained from Benzon Research, Inc. (Carlisle, PA), who established their colony in 1995 from a preexisting colony maintained at that time at Virginia Polytechnic Institute and State University. Mosquitoes were cured of *Wolbachia* infection by feeding adults for one week on 1 mg/ml tetracycline (pH 7) in 10% sucrose [67]. Mosquitoes were then fed a blood meal and maintained for one week on 10% sucrose without tetracycline before collecting eggs, followed by a second blood meal and subsequent egg collection. Mosquitoes produced from both blood meals were pooled, and used to start the *Wolbachia*(−) strain. Removal of the *Wolbachia* was confirmed by PCR.

### PCR

Total DNA was isolated from five pools of five animals each pool for *D. melanogaster* and ten pools of two animals each pool for *Cx. quinquefasciatus* (Gentra, Qiagen). The presence of *Wolbachia wsp* gene sequences and mitochondrial *12S rRNA* gene sequences was determined in each extract by PCR. Primers 81F and 691R were used for the *wsp* gene, and primers 12Sai and 12Sbi were used for the *12S* gene, both as described previously [12], [68].

### Viruses

The WNV stock was derived from WNV NY003356, a primary isolate from kidney tissue of an American crow collected in 2000 in Staten Island, NY [69]. The virus stock was prepared by three rounds of plaque purification in Vero cells. The CHIKV stock was derived from human isolate HIMTSSA 287 originally collected in the Central African Republic in 1995 and maintained at the Centers for Disease Control and Prevention, Fort Collins, CO. The virus was passaged three times in Vero cells before stock preparation. The LACV stock was likely derived from mosquito pool 74-32813 collected from New York state in 1974 and maintained at the Wadsworth Center, New York State Department of Health, Albany, NY. The virus was passaged once in BHK cells, and twice in Vero cells before stock preparation. All experiments involving infectious WNV, CHIKV, or LACV were done in the Wadsworth Center's ACL-3 laboratories.

### Virus Inoculation into *D. melanogaster*

*D*. *melanogaster* were inoculated essentially as described previously [42]. Briefly, female flies 3–5 days old were anesthetized on ice and injected intra-abdominally with ∼100 nl of Dulbecco's modified Eagle medium containing virus at the selected concentration. The injection volume was controlled with a pneumatic injector. Flies being compared within any single experiment were always injected during the same injection session, using the same injector settings and reagents, and the inoculated flies were incubated together at 27°C, 55% RH with a 16∶8 L∶D photoperiod for 7 days before being harvested for analysis of virus titer. Individual flies were placed into 0.5 mL mosquito diluent (MD: Dulbecco's phosphate-buffered saline supplemented with 20% fetal bovine serum (FBS), 50 µg/ml penicillin, 50 µg/ml streptomycin, 50 µg/ml gentamicin, 2.5 µg/ml Fungizone), homogenized using a mixer mill, and stored at −70°C until virus titers were measured by plaque assay.

### Vector competence of *Cx. quinquefasciatus*

Vector competence assays were performed essentially as described previously [70]. Briefly, 5–7 day old females were fed a blood meal of goose blood supplemented with 2.5% sucrose plus WNV at a final titer of 4×10^8^ pfu/mL. Mosquitoes were fed for 1 hourr using a Hemotek membrane feeder (Discovery Workshops, Accrington, UK). Virus titer in the blood meal did not change during the course of the 1 hour feeding. Fully engorged mosquitoes were sorted into pint cartons supplied with 10% sucrose *ad libitum* and held at 27°C, 55% RH, and 16∶8 L∶D photoperiod before being assayed. At 5, 7, and 14 days post blood meal, mosquitoes were anesthetized with triethylamine, and their legs were removed and placed into 0.5 mL MD. Saliva was collected by placing the proboscis into a capillary tube containing 50% FBS, 50% sucrose for 30 minutes, and then the solution in the capillary was dispensed into 0.5 mL MD. The body was placed into 0.5 mL MD, and the body and legs were homogenized by mixer mill. Samples were stored at −70°C until the proportion of mosquitoes with infected bodies (infected), infected legs (disseminated), and infected saliva (transmitted) was determined by plaque assay. Results obtained for the *Wolbachia*(+) and *Wolbachia*(−) strains of *Cx. quinquefasciatus* were compared using binomial probability analysis.

### Plaque Assays

Vero cell plaque assays were used to determine titers of WNV, CHIKV, and LACV essentially as described previously [71]. Virus titers in different strains of *D. melanogaster* were compared using Student's t-tests, and ID_50_ values were calculated using program ID50 5.0 [72]. Virus titers in *Wolbachia*(+) and *Wolbachia*(−) *Cx. quinquefasciatus* were compared using ANOVA analysis after confirming the data were normally distributed using the Anderson-Darling test statistic. Correlation of the probability of virus dissemination into the legs and of virus transmission into the saliva with virus titer measured in the bodies of *Wolbachia*(+) and *Wolbachia*(−) *Cx. quinquefasciatus* was done using χ^2^ analysis.

## Supporting Information

Text S1 Supplemental data for figures S1-S5.
(0.04 MB DOC)

Figure S1 *D. melanogaster* strains Oregon R and *Ago2^414^* differ in their susceptibility to WNV infection. The indicated pfu of WNV was injected into *D. melanogaster* strains wild-type Oregon R (OR) and *Ago2^414^* (414). Seven days after inoculation, the titer of WNV in each fly was measured by plaque assay. (A) The fraction of flies that became infected for each genotype at each concentration of virus, and the ID_50_ value for each genotype as calculated from those data, are shown. (B) The titers of WNV in the infected OR (O) and 414 flies (X) are shown. The grey diagonal line indicates the amount of WNV inoculated per fly. The limit of detection of the plaque assay was 25 pfu/animal for strain OR and 2.5 pfu/animal for strain 414.
(0.07 MB PDF)

Figure S2 The WNV resistance phenotype observed in *Ago2^414^* flies is a dominant maternal-effect phenotype. The indicated pfu of WNV was injected into progeny from the reciprocal crosses female OR x male 414 (MAT-OR) and female 414 x male OR (MAT-414). Seven days after inoculation, the titer of WNV in each fly was measured by plaque assay. (A) The fraction of flies that became infected for each genotype at each concentration of virus, and the ID_50_ value for each genotype as calculated from those data, are shown. (B) The titers of WNV in the infected MAT-OR (O) and MAT-414 flies (X) are shown. The grey diagonal line indicates the amount of WNV inoculated per fly. The limit of detection of the plaque assay was 25 pfu/animal for MAT-OR and 2.5 pfu/animal for MAT-414.
(0.06 MB PDF)

Figure S3 The WNV resistance phenotype observed in *Ago2^414^* flies is caused by a maternal cytoplasmic factor. Twenty three pfu of WNV was injected into female progeny from each generation of five consecutive introgression backcrosses of female progeny to OR males, starting with the cross of resistant strain 414 females to susceptible OR males. As a positive control at each generation, WNV was also injected into females from the OR stock, and the inoculated females were assayed in parallel with the female progeny from the introgression backcrosses. Seven days after inoculation, the titer of WNV was measured by plaque assay in the backcross progeny flies (X) and control OR flies (O). The ratio of the number of flies infected to the number of flies inoculated for each generation is shown along the top of the graph for the OR control flies and along the bottom of the graph for the backcross progeny flies. The limit of detection of the plaque assay was 25 pfu/animal and is shown by a dashed grey line.
(0.04 MB PDF)

Figure S4 The *Wolbachia* status of *D. melanogaster* strains analyzed for susceptibility to arbovirus infection. DNA was isolated from *D. melanogaster* strains Oregon R (OR), *Ago2^414^* (414) and tetracycline-treated *Ago2^414^* (414-T). DNA sequences corresponding to the *wsp* gene of *Wolbachia* and the *12S* mitochondrial gene were identified by PCR.
(0.09 MB PDF)

Figure S5 The WNV resistance phenotype observed in *Ago2^414^* flies was lost after tetracycline treatment. The indicated pfu of WNV was injected into *D. melanogaster* strain *Ago2^414^* (414) and tetracycline-treated *Ago2^414^* (414-T). Seven days after inoculation, the titer of WNV in each fly was measured by plaque assay. (A) The fraction of flies that became infected for each genotype at each concentration of virus, and the ID_50_ value for each genotype as calculated from those data, are shown. (B) The titers of WNV in the infected 414 (X) and 414-T flies (O) are shown. The grey diagonal line indicates the amount of WNV inoculated per fly. The limit of detection of the plaque assay was 5 pfu/animal.
(0.06 MB PDF)
