# Supplementary material for: The Native Wolbachia Endosymbionts of Drosophila melanogaster and Culex quinquefasciatus Increase Host Resistance to West Nile Virus Infection
Source: PLoS One. 2010 Aug 5;5(8):e11977. doi: 10.1371/journal.pone.0011977 (PMC2916829; doi:10.1371/journal.pone.0011977)
Supplement: Text S1 — Supplemental data for figures S1-S5. (0.04 MB DOC) [file pone.0011977.s001.doc]

**Supplemental Text**

The RNAi mutant strain *Ago2^414^* was highly resistant to infection by WNV (Fig. S1). Wild-type Oregon R flies (OR) were susceptible to WNV infection, having an ID_50_ for WNV of 2.7 pfu and consistently high virus titers 7 days after inoculation at all concentrations of virus tested. In contrast, *Ago2^414^* flies (hereafter referred to as strain 414) had an ID_50_ for WNV of 1400 pfu, more than 500-fold higher than that measured in OR flies. While injection of increasing amounts of virus could eventually produce infected strain 414 flies, virus titers were significantly lower than those observed in infected OR flies, except at the very highest WNV concentration tested. It required inoculating strain 414 flies with 100,000-fold more WNV to produce virus titers comparable to what was observed in OR flies. Compound heterozygous flies containing two different null mutations in the *Ago2* gene had higher titers of WNV during infection than wild-type flies, consistent with the RNAi pathway normally inhibiting WNV infection and demonstrating that the resistance phenotype observed in homozygous strain 414 flies was not a consequence of the loss of Ago2 activity. The same resistance phenotype was observed in female and male flies (data not shown).

The WNV resistance phenotype was transmitted as a dominant, maternal-effect trait (Fig. S2). Genetically identical female progeny from the reciprocal crosses (female OR x male 414) and (female 414 x male OR) were compared for their susceptibility to WNV infection. The phenotype of the progeny reflected the phenotype of the mother, such that strain 414 mothers produced progeny that were resistant to infection, while OR mothers produced progeny that were susceptible to infection.

The WNV resistance phenotype was caused by a maternally transmitted cytoplasmic factor in the egg and not by a maternal-effect mutation in a nuclear gene (Fig. S3). The resistance phenotype was assayed in female progeny from each generation of five consecutive introgression backcrosses of female progeny to OR males, starting with the cross of resistant strain 414 females to susceptible OR males. During the introgression backcrosses, cytoplasmic factors originating in the eggs of strain 414 females were unchanged at each generation, assuming efficient maternal transmission of any putative cytoplasmic factor, while the nuclear genome was diluted from 100% to 3% strain 414. So, in general, fifth generation flies had OR nuclear genes in a strain 414 cytoplasm. The resistance phenotype was undiminished in female progeny from the F1 through the F5 generation, despite the change in nuclear genome content. Inoculations of females from the susceptible OR strain done in parallel at each generation produced the expected levels of infection, thereby providing positive controls for inoculation and infection at each generation.

*Ago2^414^* flies are infected by *Wolbachia* (Fig. S4). DNA was isolated from strain 414 and OR flies, and the presence of *Wolbachia* *wsp* gene sequences was determined by PCR. *wsp* gene sequences were present in strain 414 but not OR flies, consistent with *Wolbachia* causing the WNV resistance phenotype. Strain 414 flies were cured of *Wolbachia* infection by growing larvae on food containing tetracycline. No *wsp* sequences were detected in DNA isolated from tetracycline-treated strain 414-T flies. Susceptibility to WNV infection was compared between the original *Wolbachia*(+) strain 414 flies and the tetracycline-treated *Wolbachia*(-) strain 414-T flies. The *Wolbachia*(+) strain 414 flies were highly resistant to WNV infection, having an ID_50_ of 1520 pfu and low virus titers when they were infected. In contrast, *Wolbachia*(-) strain 414-T flies were highly susceptible to infection, having an ID_50_ of 2 pfu and consistently high virus titers, titers comparable to those measured in *Wolbachia*(-) OR flies.
