# Supplementary material for: The Native Wolbachia Endosymbionts of Drosophila melanogaster and Culex quinquefasciatus Increase Host Resistance to West Nile Virus Infection
Source: PLoS One. 2010 Aug 5;5(8):e11977. doi: 10.1371/journal.pone.0011977 (PMC2916829; doi:10.1371/journal.pone.0011977)
Supplement: Figure S1 — D. melanogaster strains Oregon R and Ago2414 differ in their susceptibility to WNV infection. The indicated pfu of WNV was injected into D. melanogaster strains wild-type Oregon R (OR) and Ago2414 (414). Seven days after inoculation, the titer of WNV in each fly was measured by plaque assay. (A) The fraction of flies that became infected for each genotype at each concentration of virus, and the ID50 value for each genotype as calculated from those data, are shown. (B) The titers of WNV in the infected OR (O) and 414 flies (X) are shown. The grey diagonal line indicates the amount of WNV inoculated per fly. The limit of detection of the plaque assay was 25 pfu/animal for strain OR and 2.5 pfu/animal for strain 414. (0.07 MB PDF) [file pone.0011977.s002.pdf]

A

WNV ID_{50} of OR and 414 strains

	pfu per inoculation							
genotype	0.06	1	9	23	380	4400	43000	ID_{50}
OR	2/24*	6/21	19/20	21/22	21/21	24/24	nd	2.7 pfu
414	nd	0/18	0/18	0/18	1/18	17/18	18/18	1400 pfu

* number infected / number inoculated

B

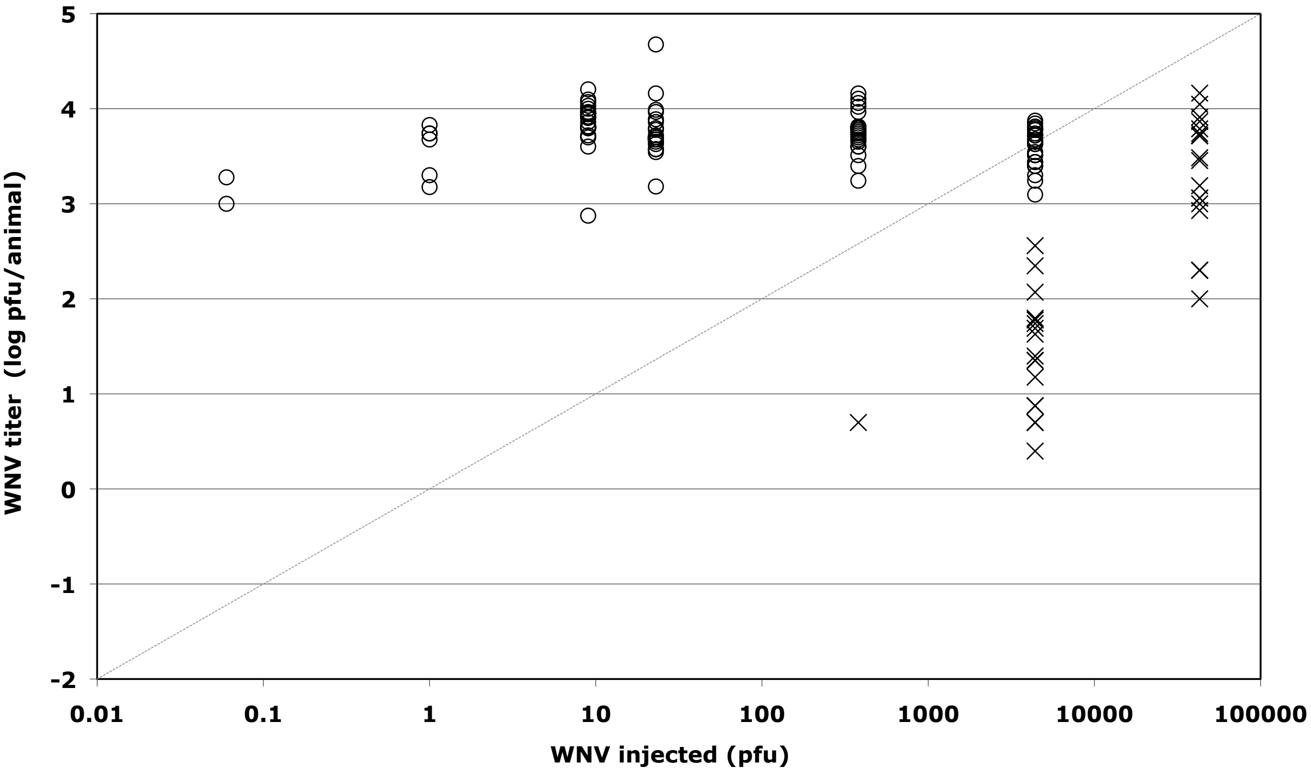

Figure S1. ***D. melanogaster*** **strains** ***Oregon R*** **and** ***Ago2*** ^{414} **differ in their susceptibility to WNV infection.**
