# Supplementary material for: The Native Wolbachia Endosymbionts of Drosophila melanogaster and Culex quinquefasciatus Increase Host Resistance to West Nile Virus Infection
Source: PLoS One. 2010 Aug 5;5(8):e11977. doi: 10.1371/journal.pone.0011977 (PMC2916829; doi:10.1371/journal.pone.0011977)
Supplement: Figure S2 — The WNV resistance phenotype observed in Ago2414 flies is a dominant maternal-effect phenotype. The indicated pfu of WNV was injected into progeny from the reciprocal crosses female OR x male 414 (MAT-OR) and female 414 x male OR (MAT-414). Seven days after inoculation, the titer of WNV in each fly was measured by plaque assay. (A) The fraction of flies that became infected for each genotype at each concentration of virus, and the ID50 value for each genotype as calculated from those data, are shown. (B) The titers of WNV in the infected MAT-OR (O) and MAT-414 flies (X) are shown. The grey diagonal line indicates the amount of WNV inoculated per fly. The limit of detection of the plaque assay was 25 pfu/animal for MAT-OR and 2.5 pfu/animal for MAT-414. (0.06 MB PDF) [file pone.0011977.s003.pdf]

A

WNV ID_{50} of MAT-OR and MAT-414 progeny

genotype	pfu per inoculation 1	9	23	380	4400	43000	ID_{50}
MAT-OR	0/16*	11/16	14/16	16/16	16/16	nd	7 pfu
MAT-414	0/16	0/16	2/17	3/17	13/13	18/18	748 pfu

* number infected / number inoculated

B

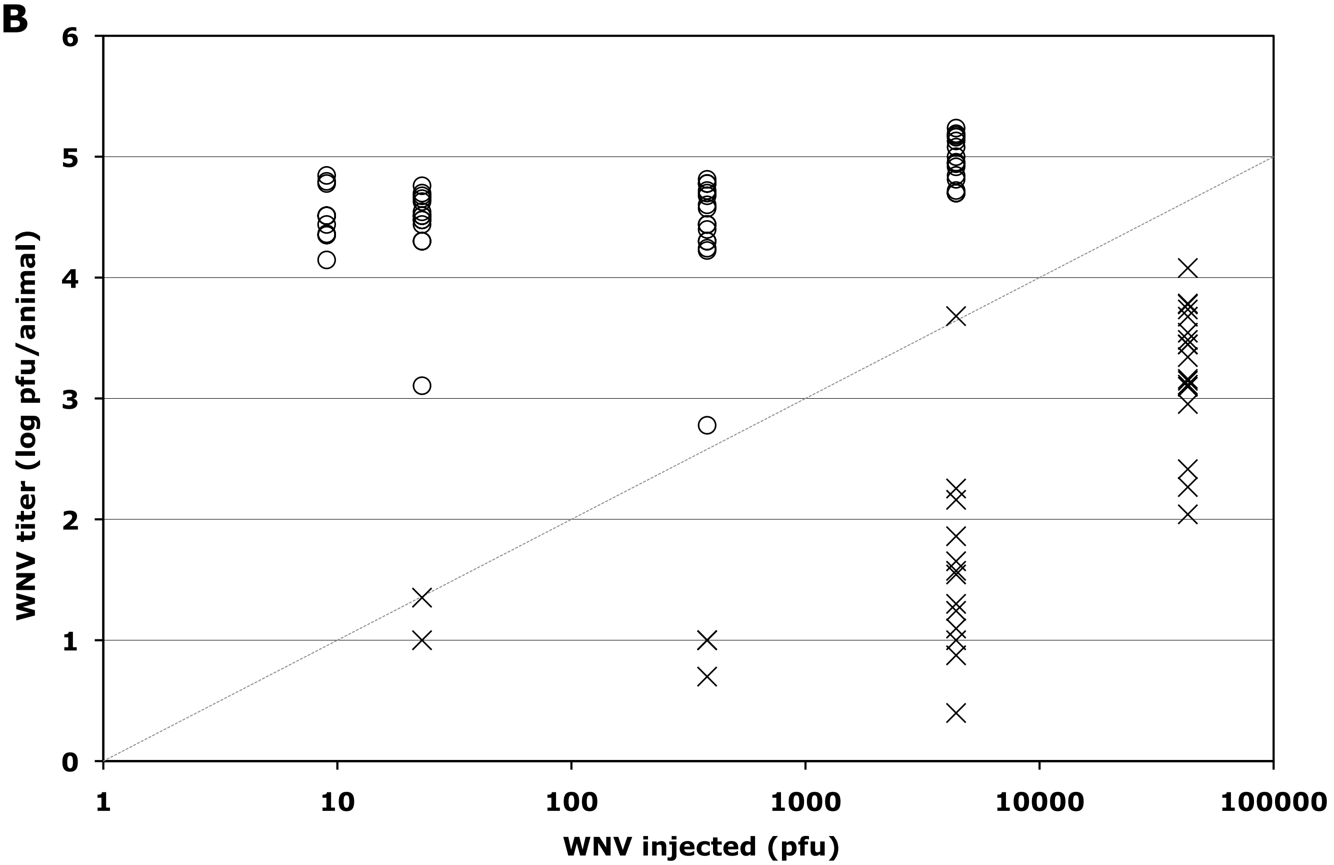

Figure S2. The WNV resistance phenotype observed in *Ago2*[414] flies is a dominant maternal-effect phenotype.
