# Supplementary material for: The Native Wolbachia Endosymbionts of Drosophila melanogaster and Culex quinquefasciatus Increase Host Resistance to West Nile Virus Infection
Source: PLoS One. 2010 Aug 5;5(8):e11977. doi: 10.1371/journal.pone.0011977 (PMC2916829; doi:10.1371/journal.pone.0011977)
Supplement: Figure S3 — The WNV resistance phenotype observed in Ago2414 flies is caused by a maternal cytoplasmic factor. Twenty three pfu of WNV was injected into female progeny from each generation of five consecutive introgression backcrosses of female progeny to OR males, starting with the cross of resistant strain 414 females to susceptible OR males. As a positive control at each generation, WNV was also injected into females from the OR stock, and the inoculated females were assayed in parallel with the female progeny from the introgression backcrosses. Seven days after inoculation, the titer of WNV was measured by plaque assay in the backcross progeny flies (X) and control OR flies (O). The ratio of the number of flies infected to the number of flies inoculated for each generation is shown along the top of the graph for the OR control flies and along the bottom of the graph for the backcross progeny flies. The limit of detection of the plaque assay was 25 pfu/animal and is shown by a dashed grey line. (0.04 MB PDF) [file pone.0011977.s004.pdf]

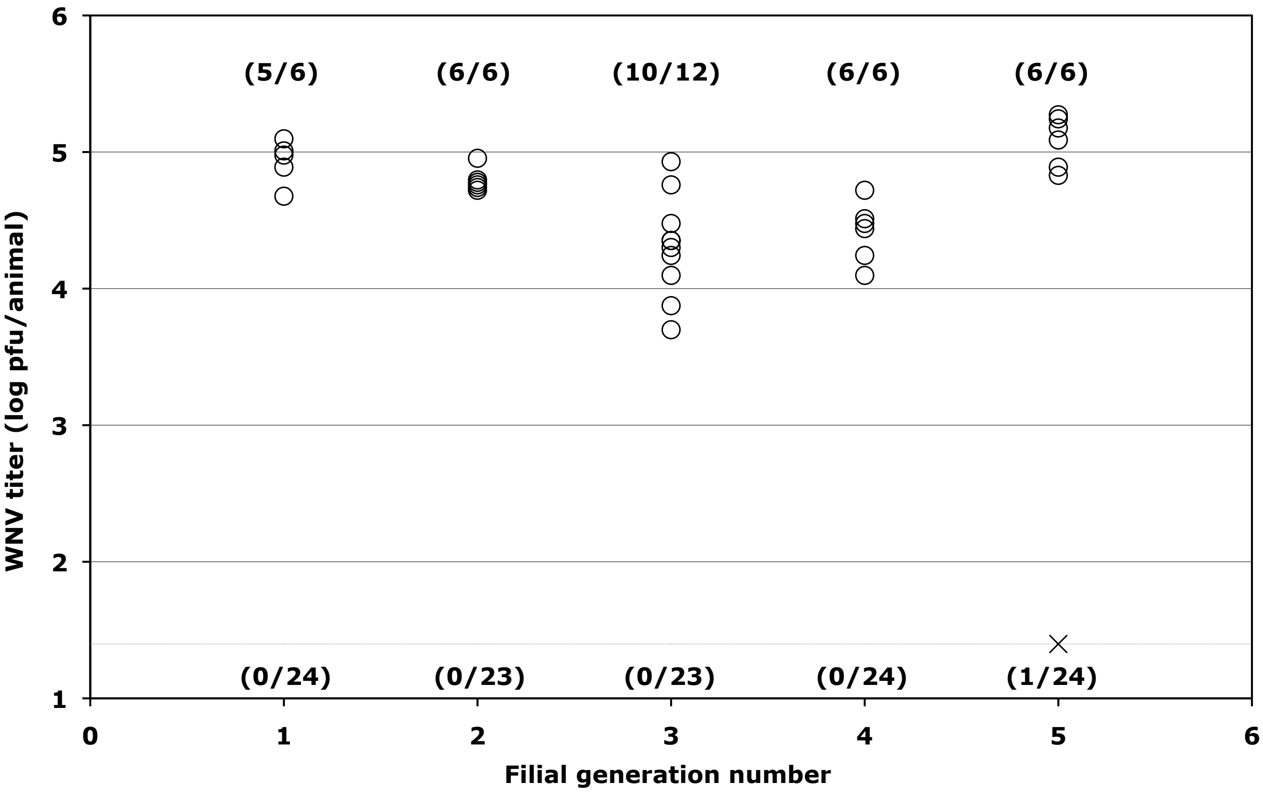

Figure S3. The WNV resistance phenotype observed in *Ago2*^{414} flies is caused by a maternal cytoplasmic factor.
