# Supplementary material for: The Native Wolbachia Endosymbionts of Drosophila melanogaster and Culex quinquefasciatus Increase Host Resistance to West Nile Virus Infection
Source: PLoS One. 2010 Aug 5;5(8):e11977. doi: 10.1371/journal.pone.0011977 (PMC2916829; doi:10.1371/journal.pone.0011977)
Supplement: Figure S4 — The Wolbachia status of D. melanogaster strains analyzed for susceptibility to arbovirus infection. DNA was isolated from D. melanogaster strains Oregon R (OR), Ago2414 (414) and tetracycline-treated Ago2414 (414-T). DNA sequences corresponding to the wsp gene of Wolbachia and the 12S mitochondrial gene were identified by PCR. (0.09 MB PDF) [file pone.0011977.s005.pdf]

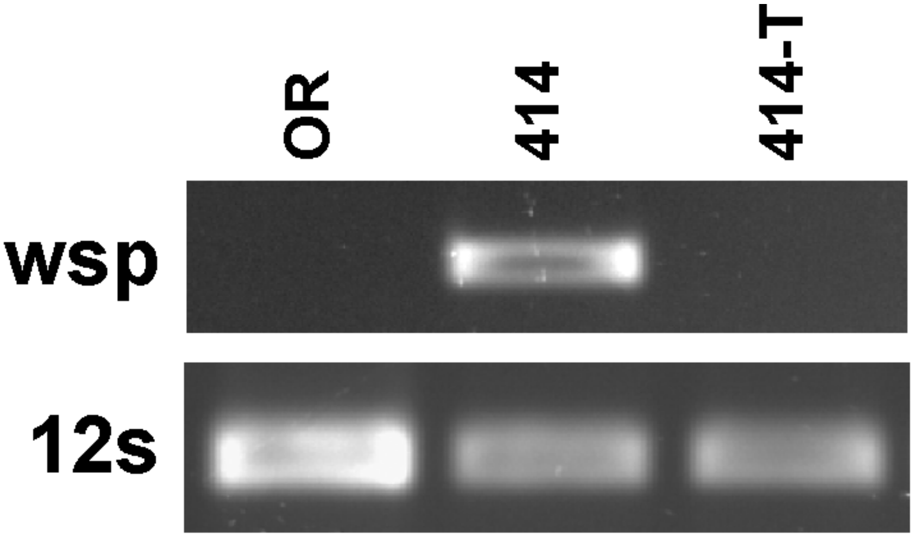

Figure S4. The *Wolbachia* status of *D. melanogaster* strains analyzed for susceptibility to arbovirus infection.
