# Supplementary material for: The Native Wolbachia Endosymbionts of Drosophila melanogaster and Culex quinquefasciatus Increase Host Resistance to West Nile Virus Infection
Source: PLoS One. 2010 Aug 5;5(8):e11977. doi: 10.1371/journal.pone.0011977 (PMC2916829; doi:10.1371/journal.pone.0011977)
Supplement: Figure S5 — The WNV resistance phenotype observed in Ago2414 flies was lost after tetracycline treatment. The indicated pfu of WNV was injected into D. melanogaster strain Ago2414 (414) and tetracycline-treated Ago2414 (414-T). Seven days after inoculation, the titer of WNV in each fly was measured by plaque assay. (A) The fraction of flies that became infected for each genotype at each concentration of virus, and the ID50 value for each genotype as calculated from those data, are shown. (B) The titers of WNV in the infected 414 (X) and 414-T flies (O) are shown. The grey diagonal line indicates the amount of WNV inoculated per fly. The limit of detection of the plaque assay was 5 pfu/animal. (0.06 MB PDF) [file pone.0011977.s006.pdf]

A

WNV ID_{50} of untreated and tetracycline-treated 414

	pfu inoculated					
genotype	1	9	23	380	4400	ID_{50}
414	1/18*	0/13	1/15	0/16	16/18	1,520 pfu
414-T	1/11	18/18	17/17	16/16	11/11	2 pfu

* number infected / number inoculated

B

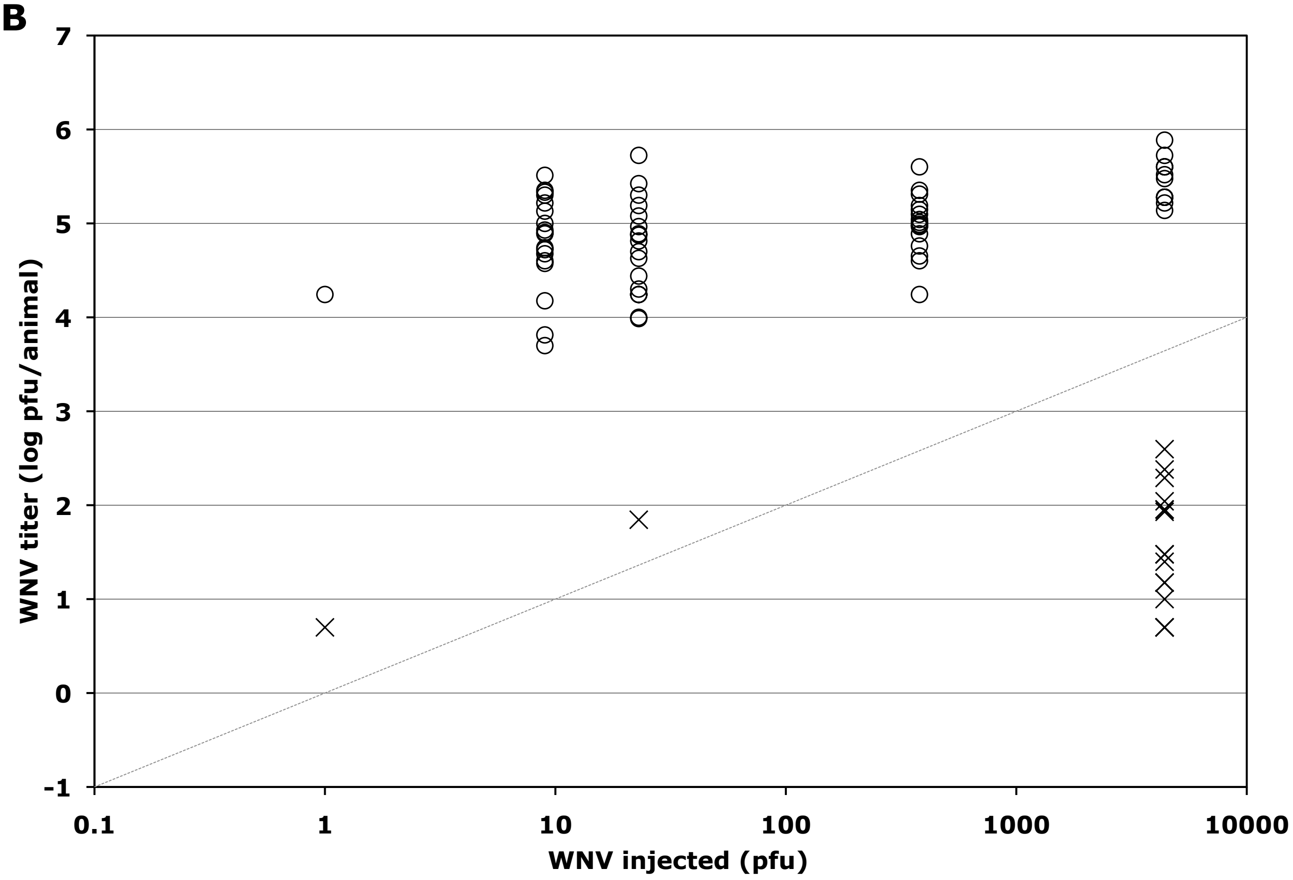

Figure S5. The WNV resistance phenotype observed in *Ago2*[414] flies was lost after tetracycline treatment.
